# Supplementary material for: Expanding the Molecular Spectrum of ANKRD11 Gene Defects in 33 Patients with a Clinical Presentation of KBG Syndrome
Source: Int J Mol Sci. 2022 May 25;23(11):5912. doi: 10.3390/ijms23115912 (PMC9180463; doi:10.3390/ijms23115912)
Supplement: Supplementary file 1 [file ijms-23-05912-s001.zip › Table S2_revised.pdf]

Table S2: ACMG criteria of the rare *ANKRD11* variants identified in the study.

Chromosome position	HGVS nomenclature*	rs number	ACMG classification#	ACMG criteria
chr16:89341329	c.7606C>T, (p.Arg2536Trp)	unreported	LP	PS2, PM1, PM2, PP3, BP1
chr16:89345919	c.7031T>C, (p.Leu2344Pro)	unreported	US	PM2, PP3, BP1
chr16:89346113	c.6836_6837delTG, (p.Val2279GlyfsTer16)	rs1555525296	P	PVS1, PS2, PM2, PP5
chr16:89346392	c.6552_6558dupTGAGGAG, (p.Pro2187Ter)	unreported	P	PVS1, PS2, PM2, PP3
chr16:89346516	c.6434C>T, (p.Thr2145Ile)	rs761862402	B	BS1, BS2, BP1, BP4
chr16:89347549	c.5401G>A, (p.Glu1801Lys)	rs938676909	LB	PM2, BP1, BP4
chr16:89347752	c.5198C>T, (p.Ala1733Val)	rs148243995	B	BP6, BS1, BS2, BP4
chr16:89348536	c.4414G>A, (p.Glu1472Lys)	rs1597451653	LB	PM2, BP1, BP4
chr16:89348689	c.4261G>T, (p.Glu1421Ter)	unreported	P	PVS1, PS2, PM2, PP3
chr16:89349181	c.3768_3769delCA, (p.His1256GlnfsTer26)	unreported	P	PVS1, PM2, PP5
chr16:89349628	c.3319_3322delAAAG, (p.Lys1107AlafsTer210)	unreported	P	PVS1, PM2, PP3
chr16:89349963	c.2987G>T, (p.Gly996Val)	rs1205687342	LB	PM2, BP1, BP4
chr16:89350427	c.2523G>A, (p.Trp841Ter)	unreported	P	PVS1, PS2, PM2, PP3
chr16:89350538	c.2408_2412delAAAAA, (p.Lys803ArgfsTer5)	rs886039902	P	PVS1, PM2, PP3, PP5
chr16:89350549	c.2398_2401delGAAA, (p.Glu800AsnfsTer62)	rs797045027	P	PVS1, PM2, PP5
chr16:89350582	c.2368G>T, (p.Glu790Ter)	unreported	P	PVS1, PM2
chr16:89350753	c.2197C>T, (p.Arg733Ter)	rs886041791	P	PVS1, PM2, PP3, PP5
chr16:89351043	c.1903_1907delAAACA, (p.Lys635GlnfsTer26)	rs886041125	P	PVS1, PS2, PM2, PP3, PP5
chr16:89351491	c.1459G>T, (p.Glu487Ter)	unreported	P	PVS1, PS2, PM2, PP3
chr16:89351563	c.1388_1389delAA, (p.Lys463ArgfsTer29)	unreported	P	PVS1, PS2, PM2, PP3
chr16:89351566	c.1381_1384delGAAA, (p.Glu461GlnfsTer48)	unreported	P	PVS1, PS2, PM2, PP3, PP5
chr16:89351566	c.1381_1384delGAAA, (p.Glu461GlnfsTer48)	unreported	P	PVS1, PS2, PM2, PP3, PP5
chr16:89351578	c.1372C>T, (p.Arg458Ter)	rs900492387	P	PVS1, PM2, PP5
chr16:89351718	c.1232C>A, (p.Ser411Ter)	unreported	P	PVS1, PM2

P, pathogenic; LP, likely pathogenic; LB, likely benign; US, uncertain significance; B, benign

*HGVS nomenclature applies to GenBank: NG_032003, GenBank: NM_013275.6, and GenBank: NP_037407

#classifications include pathogenic (P), likely pathogenic (LP), uncertain significance (US), likely benign (LB), and benign (B) variants
