# Supplementary material for: Expanding the Molecular Spectrum of ANKRD11 Gene Defects in 33 Patients with a Clinical Presentation of KBG Syndrome
Source: Int J Mol Sci. 2022 May 25;23(11):5912. doi: 10.3390/ijms23115912 (PMC9180463; doi:10.3390/ijms23115912)
Supplement: Supplementary file 1 [file ijms-23-05912-s001.zip › Table S3.pdf]

Table S3. Statistical analysis between the main KBG clinical signs of KBGS confirmed and not confirmed patients

**Clinical signs**	**KBG_pos**	**KBG_neg**	**noKBG_pos**	**noKBG_neg**	**% of KBG confirmed patients**	**% of not confirmed patients**	**P value Fisher**
Stature below 10th centile	12	10	4	7	55	36	0,465
Microcephaly	4	18	2	9	18	18	1,000
Triangular face	13	8	5	6	62	45	0,465
Low anterior and/or posterior hairline	13	7	2	8	65	20	0,050
Broad bushy eyebrows	15	5	7	4	75	64	0,683
Synophrys	13	7	3	8	65	27	0,066
Prominent nasal bridge	12	9	6	5	57	55	1,000
Anteverted nares	11	10	4	7	52	36	0,472
Long smooth philtrum	12	8	9	2	60	82	0,262
Thin upper lip	12	9	7	1	57	88	0,201
Palatal defects	4	15	4	7	21	36	0,417
Macrodontia (upper central incisors)	13	4	7	3	76	70	1,000
Large anterior fontanelle and/or delayed closure	6	5	1	4	55	20	0,308
Vertebral anomalies	1	7	1	2	13	33	0,491
Fifth fingers clinodactyly	9	13	3	8	41	27	0,703
Brachydactyly	13	8	4	7	62	36	0,266
Developmental delay	18	2	10	1	90	91	1,000
Learning difficulty	19	0	9	1	100	90	0,345
Seizures	7	14	4	7	33	36	1,000
Behavioural abnormalities	11	10	6	5	52	55	1,000
Hearing loss	14	6	3	7	70	30	0,056
Cryptorchidism	6	5	3	4	55	43	1,000
Feeding problems	8	11	3	4	42	43	1,000

KBG_pos, number of KBG confirmed patients showing the clinical sign; KBG_neg, number of KBG confirmed patients without the clinical sign; noKBG_pos, number of not confirmed KBG patients showing the clinical sign; noKBG_neg, number of not confirmed KBG patients without the clinical sign
