# Supplementary material for: Expanding the Molecular Spectrum of ANKRD11 Gene Defects in 33 Patients with a Clinical Presentation of KBG Syndrome
Source: Int J Mol Sci. 2022 May 25;23(11):5912. doi: 10.3390/ijms23115912 (PMC9180463; doi:10.3390/ijms23115912)
Supplement: Supplementary file 1 [file ijms-23-05912-s001.zip › Table S5_revised.pdf]

Table S5. Primers used for CNVs molecular characterizations

Designation		Primer sequence (5'--> 3')	Primer localization*	Amplicon size	TA	Application
PT4_probe_ex12	Fw	CAAGTATGACCGCATGAAGG	chr16:89337224-89337243	78 bp	60°C	qPCR analysis
	Rev	ATCACAGAACTGGGCAGCA	chr16: 89337166-89337185			
PT14_probe1	Fw	CCGCCTGCTCTCATTTCA	chr16:89555954-89555971	69 bp	60°C	qPCR analysis
	Rev	CCGTGCTGGTCGTAGTTGTT	chr16:89556003-89556022			
PT14_probe2	Fw	AGCGCCGTGAGTTAGTGC	chr16:89557095-89557112	67 bp	60°C	qPCR analysis
	Rev	GACCGCCAGAGAGGCTGT	chr16: 89557144-89557161			
PT14_probe3	Fw	AGGTGAACAAAATCGGTGCT	chr16:89557856-9557875	60 bp	60°C	qPCR analysis
	Rev	AGTCCCCAGTAACATGCAGAA	chr16:89557895-89557915			
PT14_probe4	Fw	GAGCGCCTGATAAAAACAGC	chr16:89558644-89558663	91 bp	60°C	qPCR analysis
	Rev	CTTCGTCCAGTGAGGCAAAT	chr16:89558715-89558734			
PT4_bkp_pcr	Fw	TTAGAAATAGTGAGGCTGTTGTGAGGTG	chr16:89300930-89300957	39342 bp	63.5°C	Bkps junction amplification
	Rev	CACTCTGAGCAGTGCACTAGGCTTT	chr16:89340247-89340271			
PT14_bkp_pcr	Fw	*PT14_probe1_Fw*		2781 bp	60°C	Bkps junction amplification
	Rev	*PT14_probe4_Rev*				

Fw, forward; Rev, reverse; TA, annealing temperatur

* Primer physical localization is based on GRCh37/hg19 human genome assembly.
